# Laparoscopic Fenestration of a Giant Pseudocyst after Totally Extra Peritoneal Inguinal Hernia Repair

**DOI:** 10.1155/2016/9867645

**Published:** 2016-11-28

**Authors:** Yu-Ting van Loon, Maaike S. Ibelings

**Affiliations:** Department of Surgery, Elisabeth-TweeSteden Hospital, Tilburg, Netherlands

## Abstract

A giant pseudocyst is a rare complication after hernioplasty and is seldom seen. The pathophysiology is unclear; it characteristically does not contain epithelial lining and can be considered giant if the diameter exceeds 10 cm. Pseudocysts are mostly described after incisional hernia repairs and are usually treated with surgical resection. We report a case of a giant pseudocyst three years after totally extra peritoneal inguinal hernia repair. Laparoscopic fenestration without removing the pseudocyst with or without removal of the polypropylene mesh is a safe and effective minimal invasive approach to the treatment of a symptomatic pseudocyst and should also be considered in the approach of other large symptomatic cysts.

## 1. Case Presentation

**Figure 1:**
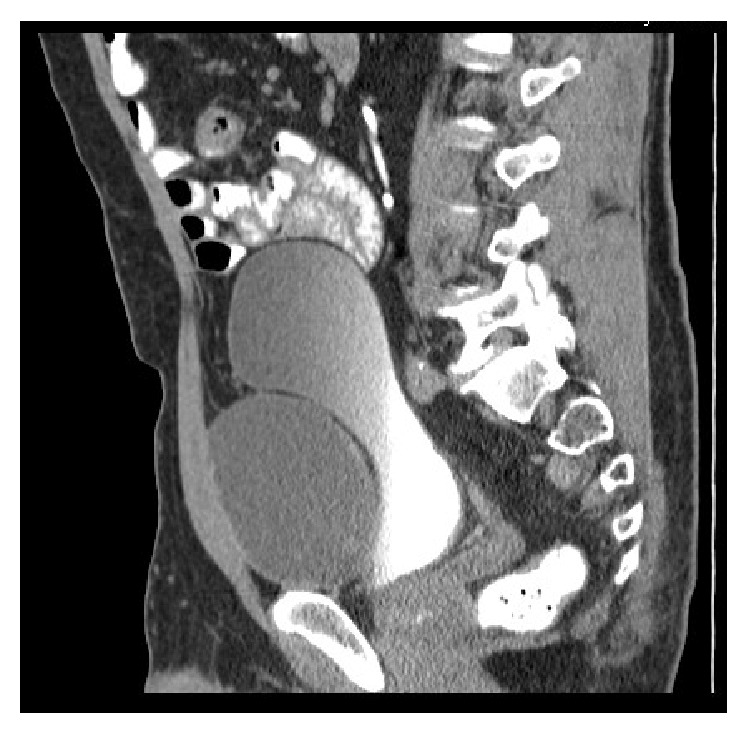
Sagittal computer tomography scan: a giant fluid collection of almost 16 centimeters in diameter is seen ventrally of the bladder in Retzius' space, evidently compressing the bladder.

**Figure 2:**
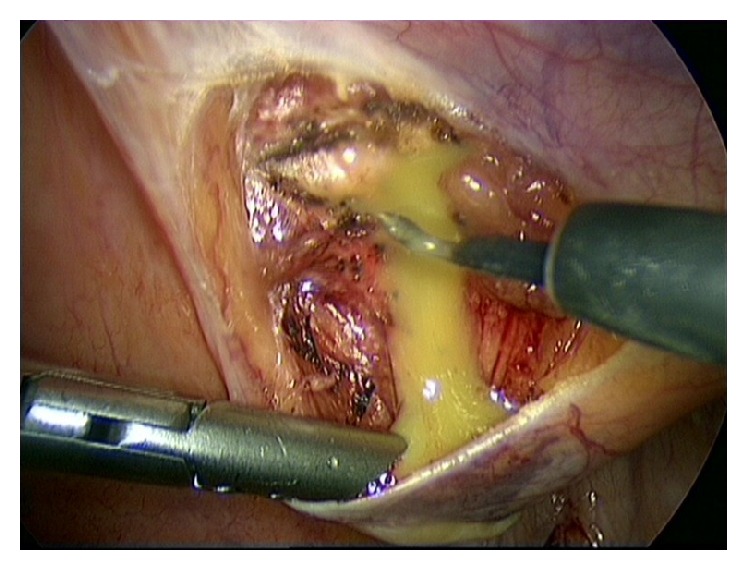
Opening of the cyst revealed approximately 600 cc of green-yellow viscous fluid.

**Figure 3:**
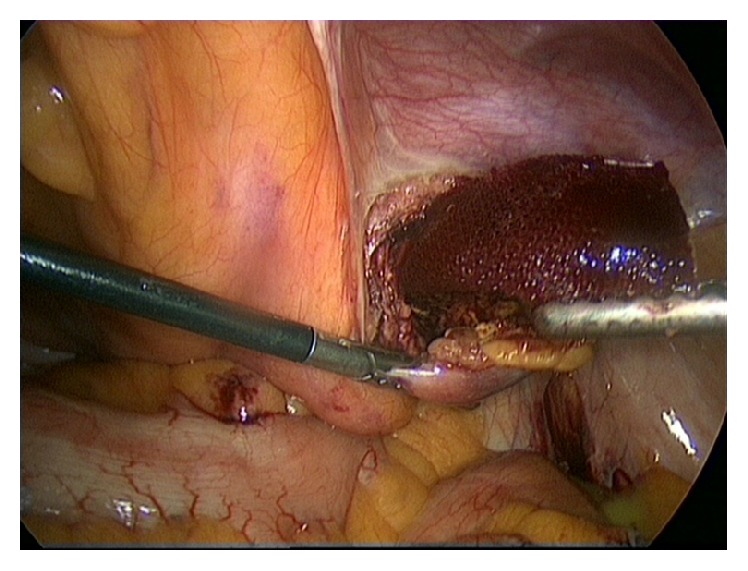
Polypropylene mesh is easily removed by simply pulling it out of the cystic cavity.

**Figure 4:**
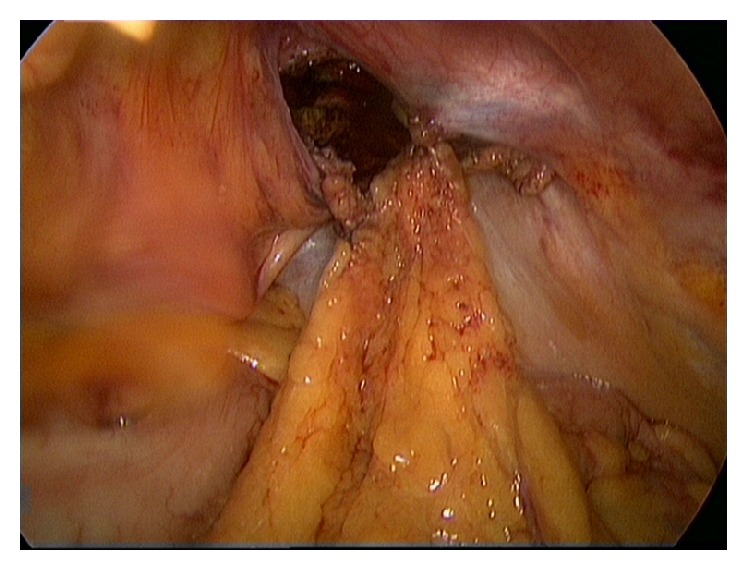
Omental patch was secured with minimal tension in the opening of the cystic cavity.

A 63-year-old healthy male patient underwent a laparoscopic bilateral totally extra peritoneal inguinal hernia repair with two polypropylene meshes in 2010. His medical history reveals no use of any medication, nor diabetes, cardiopulmonary, or other diseases. He had stopped smoking 20 years ago and has a normal body mass index. The surgery went well and according to Dutch practice no tacks were placed. His postoperative recovery was uncomplicated; there were no signs of seroma, hematoma, or recurrence. After almost three years, the patient returned with complaints of swelling in the left inguinal area and lower urinary tract symptoms. The urinary problems were initially successfully treated with an alpha-1-antagonist. Ultrasound and computer tomography (CT) showed a cyst-like fluid collection, approximately 6 cm in diameter. Ultrasound guided aspiration of the cyst was performed due to progressive complaints of restriction of abdominal movement. The cysts' content was a clear serous liquid. Due to persisting urinary problems, a cystoscopy was performed three months later. It showed an abnormal indentation on the ventral side of the bladder, indicative of external bladder compression. CT showed a giant fluid collection of almost 16 centimeters in diameter ventrally of the bladder in Retzius' space, evidently compressing the bladder and causing the urinary problems (Figure 1). After extensive interdisciplinary deliberation with many experts in Netherlands in the field of inguinal hernia repair, the most minimally invasive approach was chosen: aspiration and sclerotherapy with a tetracycline solution. This time, the cystic content was a brown viscous liquid. Cultures showed no signs of bacterial growth. After initial success, the patient returned with the same symptoms, reappearing after just one week. Ultrasound showed an image of “flying” mesh within a recurrent cystic cavity, which had taken on the original dimensions in less than three weeks. We chose to perform a laparoscopic fenestration due to the localization and the extent of the cyst. Opening of the cyst revealed approximately 600 cc of green-yellow viscous fluid and two floating polypropylene meshes, which could be easily removed by simply pulling them out of the cystic cavity (Figures 2 and 3). The cystic cavity was cleaned and roughened. Subsequently, an omental patch was secured with minimal tension in the opening of the cystic cavity, which was left in site (Figure 4). The fluid and polypropylene mesh were sent for cultures and a piece of the cystic wall was sent for histopathological examination. The patient was discharged the next day and made an excellent recovery. Even though the cystic content appeared similar to pus, cultures showed no growth of bacteria. Histopathology showed that the cystic wall consisted of connective tissue with chronic inflammation without epithelial lining, confirming the diagnosis of a pseudocyst. There was no sign of recurrence of the pseudocyst during the last follow-up, three years after the laparoscopic fenestration. Unfortunately ultrasound showed bilateral recurrence of the inguinal hernia. He chose to refrain from further surgery since the symptoms of the inguinal hernia were not troublesome or problematic in his daily activities.

## 2. Discussion

Inguinal hernia repair is one of the most commonly performed surgical procedures worldwide. Particularly placement of polypropylene meshes in hernia repair has become the acknowledged and standard surgical treatment. Postoperative seroma, hematoma, and wound infection are well known, early complications [1–4]. Formation of a giant pseudocyst is a rare complication after hernioplasty [4, 5] with an estimated prevalence of 0.45–0.88% based on two clinical researches [6] and is therefore seldom seen. A pseudocyst, mature fibrous cyst or cystic seroma characteristically has no epithelial lining in contrary to a “regular” cyst and can be considered giant if the diameter exceeds 10 centimeters [7]. The pathophysiology is still unclear; most authors support the hypothesis that a pseudocyst could be the result of seroma or hematoma formation. Some authors even question this entity to be an underreported complication, since seroma and hematoma are so frequently observed [2, 3, 5, 7].

The majority of the case reports discuss pseudocyst after incisional hernia repairs [1, 2, 5–9]; this is the second case report worldwide as a result of inguinal hernia repair and the first case report mentioning laparoscopic fenestration without removal of the pseudocyst. Our first attempt at treating the pseudocyst by aspiration and sclerotherapy failed. Aspiration therapy is considered fruitless and palliative in literature since it is difficult to perform due to septums or trabeculations in the cyst or the thick detritus cystic content and it always recurs [2, 8]. Tetracycline sclerotherapy is mentioned in the literature as therapy for ovarian cysts, hydroceles, and renal cysts. The efficacy of sclerotherapy lies in the biological potency of the sclerosant to permanently disrupt the endothelium of vascular structures, endothelial lining of cysts, capillary beds, or lymphatic structures which causes an inflammatory reaction resulting in fibrosis [9]. We believe that the tetracycline sclerotherapy failed in our patient because of the absence of epithelial lining in the pseudocyst. The available literature on pseudocysts mentions a surgical treatment without removal of the mesh, due to the incorporation of the mesh into the cyst or abdominal wall [1, 2, 6–8, 10]. Leaving the mesh in situ also ensures the integrity of the hernioplasty and therefore possible reduction of the risk of future recurrence. We chose laparoscopy because of the localization of the cyst combined with our desire for a minimally invasive approach. To our surprise, however, we found both of the meshes floating freely in the cystic content within the pseudocyst. There were no signs of adhesions or fibrosis with the cystic wall or abdominal muscles. Fenestration of the pseudocyst and placement of an omental patch was meant to secure a permanent outlet for and absorption of the cystic content, preventing it from retaining or forming a new giant symptomatic pseudocyst.

To the best of our knowledge this is the first case report describing a laparoscopic fenestration as a successful treatment of a pseudocyst. We believe that it is a safe, effective, and patient friendly approach and should be considered before a major laparotomy if the abdomen is laparoscopically accessible.

## 3. Conclusion

A pseudocyst is a rare complication after hernioplasty, possibly as a result of seroma or hematoma formation. Aspiration and sclerotherapy are fruitless; the best treatment described so far is surgical resection of the pseudocyst. Laparoscopic fenestration without removing the pseudocyst with or without removal of the polypropylene mesh is a safe and effective minimal invasive approach to the treatment of a symptomatic pseudocyst and should also be considered in the approach of other large symptomatic cysts.
